# ALKBH5 inhibits TNF-α-induced apoptosis of HUVECs through Bcl-2 pathway

**DOI:** 10.1515/med-2022-0484

**Published:** 2022-06-15

**Authors:** Xiaoshan Zhang, ShiBing Deng, Yang Peng, Han Wei, Zhiming Tian

**Affiliations:** Department of Cardiology, First School of Clinical Medicine College, Yangtze University, Hubei, China; Department of Cardiology, First School of Clinical Medicine College, Yangtze University, No. 8, Hangkong Road, Jingzhou 434000, Hubei, China

**Keywords:** ALKBH5, apoptosis, atherosclerosis, N6-methyladenosine, Bcl-2

## Abstract

The dysfunction and apoptosis of vascular endothelial cells are the initiating links in the formation of atherosclerosis. N6-methyladenosine (m6A) is an extremely extensive RNA methylation modification and its abnormality leads to the occurrence of various human diseases. In this study, we explored the effects of demethylase *α*-ketoglutarate-dependent dioxygenase ALKB homolog 5 (ALKBH5) on TNF-α-induced apoptosis of human umbilical vein endothelial cells (HUVECs). In TNF-α-treated HUVECs, the expression of ALKBH5 was significantly decreased. ALKBH5 overexpression promoted the proliferation and inhibited the apoptosis in TNF-α-treated HUVECs, suggesting that ALKBH5 had a protective effect on cell damage induced by TNF-α. Importantly, ALKBH5 promoted the expression of Bcl-2 in HUVECs. Bcl2 overexpression reduced the expression of Gadd45, Bax, and p21, which are transcriptionally activated by p53. But the expression of p53 has not been significantly affected, indicating that Bcl2 might regulate the apoptosis by inhibiting p53 downstream targets. In addition, ALKBH5 overexpression significantly increased the level of pri-miR-7 and decreased the level of miR-7. In conclusion, ALKBH5 attenuated the TNF-α-induced cell injury via promoting Bcl2 expression. Our research expands the understanding of the progression mechanism of atherosclerosis and provides a potential strategy for the protection of vascular endothelial injury.

## 1 Introduction

Atherosclerosis (AS) is a systemic or local inflammatory and immunoreactive disease mediated by inflammatory factors and chemokines. The inflammatory damage, dysfunction, and apoptosis of vascular endothelial cells are the initiating links in the formation of AS [1]. Therefore, exploring strategies to protect endothelial cells is of vital importance for the prevention and treatment of AS.

N6-methyladenosine (m6A) is an extremely extensive RNA methylation modification that participates in the transport, shearing, translation, and processing of mRNA [2,3,4]. Its abnormality often leads to changes in the corresponding downstream target proteins, which leads to the occurrence of diseases [2,5]. *α*-Ketoglutarate-dependent dioxygenase ALKB homolog 5 (ALKBH5) is a primary m6A demethylase, a member of the AlkB family, and also a homolog of obesity-related genes (FTO) [6]. At present, ALKBH5 is found to play a biological and pharmacological role in human cancer or non-cancer diseases [6,7]. ALKBH5 participates in the regulation of the occurrence and metastasis of human tumors by regulating a variety of biological processes, such as proliferation, migration, invasion, and metastasis [7,8]. In addition, ALKBH5 also plays an important role in the reproductive system diseases and bone metabolism [9,10,11]. Studies have shown that m6A methylation is active in vascular endothelial cells. m6A methylation is involved in the pathologies of myocardial ischemia-reperfusion injury and AS, including oxidation, stress, inflammation, and metabolic disorders [12,13]. Currently, the functions of three m6A methylation (METTL3, METTL14, and WTAP) and demethylases FTO in vascular endothelial dysfunction have been reported [14,15,16]. However, the role of ALKBH5 in vascular endothelial injury and AS remains unknown. Here we aimed to explore the specific effect of ALKBH5 on vascular endothelial cells.

In many studies, TNF-α-treated human umbilical vein endothelial cells (HUVECs) were used to simulate arteriosclerosis *in vitro*. Here we explored the effects of ALKBH5 on TNF-α-induced apoptosis of HUVECs and its specific pathways in an *in vitro* cell model, trying to provide a potential strategy for the protection of vascular endothelial injury.

## 2 Methods

### 2.1 Cell culture and treatment

HUVECs were purchased from KeyGen BioTECH Corp., Ltd (Nanjing, China) and grown in DMEM (11995065, Thermo Fisher Scientific, San Jose, USA) with 5% CO_2_ at 37℃. The model of endothelial cell injury was constructed with 10 ng/mL TNF-α for 24 h. Overexpression plasmids, pcDNA3.1-ALKBH5 and pcDNA3.1-Bcl2 were purchased from YouBio (Changsha, China). The siRNA-Bcl2 and siRNA-control were synthetized from Sangon Biotech (Shanghai, China). Lipofectamine 2000 (11668500, Thermo Fisher Scientific, San Jose, USA) was used for the transfection. miR-7 mimic (5′-TGGAAGACTAGTGATTTTGTTG-3′) and negative control (NC) mimic (5′-GGUUCGUACGUACACUGUUCA-3′) were synthetized from GeneChem Co., Ltd (Shanghai, China).

### 2.2 Western blot assay

Total protein was extracted from HUVECs after the treatment or transfection for 24 h using a radio immunoprecipitation assay (RIPA, R0010, Solarbio Science and Technology Ltd, Beijing, China) buffer. Sodium dodecyl sulfate polyacrylamide gel electrophoresis (SDS-PAGE) was performed to separate protein samples. Then, the protein was transferred into a PVDF membrane. After being blocked with non-fat milk for 1 h, the membrane was incubated with the primary antibody for 1 h, and then with the secondary antibody for 1 h at room temperature. The protein was immunoluminescent with ECL kit (CW0048M, CoWin Biotech Co. Ltd, Beijing, China). Antibodies against ALKBH5 (1:1,000, ab195377), Bcl2 (1:1,000, ab32124), p21 (1:2,000, ab109520), Bax (1:2,000, ab32503), and Gadd45 (1:500, ab205252) were purchased from Abcam (Cambridge, UK).

### 2.3 Quantitative real-time PCR (qRT-PCR)

Total mRNA was extracted from HUVECs using miRNA Purification Kit and Ultrapure RNA Kit (CW0627 and CW0581, CoWin Biotech Co. Ltd, Beijing, China). Reverse transcription was performed using cDNA Synthesis Kits (CW2141 and CW2569, CoWin Biotech Co. Ltd, Beijing, China). Then, the expression of miRNA was detected using qPCR Assay Kits (CW2142 and CW0957, CoWin Biotech Co. Ltd, Beijing, China). Relative expression levels were calculated using 2^−ΔΔCT^ method. Primers for Bcl-2 (sense, 5′-AGACTCTGCTCAGTTTGGCC-3′; antisense, 5′-TCACTTGTGGCCCAGATAGG-3′), miR-7 (5′-TGGAAGACTAGTGATTTTGTT-3′) and pre-miR-7 (5′-CGCTTCGTGTTGTATGGAAGAC-3′) were used in this research.

### 2.4 CCK8 assay

Cell viability was detected using CCK8 assay. After the transfection or treatment, cells were seeded into a 96-well plate at a density of 5000 cells/well. Cell viability was measured every 24 h. 10 µL of CCK8 reagent (CA1210, Solarbio Science and Technology Ltd, Beijing, China) was added to the cells and maintained for 1.5 h at 37°C. The cell absorbance was measured at 450 nm using a spectrophotometer.

### 2.5 Flow cytometry

The apoptosis was detected using Flow cytometry analysis. After the transfection or treatment for 24 h, cells were incubated with Annexin V/FITC (Thermo Fisher Scientific, San Jose, USA) at room temperature for 5 min and incubated with PI at room temperature for 15 min. FlowJo software was used to analyze the flow results.

### 2.6 Statistical analysis

Data in this research was presented as mean value ± standard deviation (SD). Statistical analysis was performed using GraphPad Prism 7 software. Differences between two groups were analyzed using the student’s *t* test, and differences between three and four groups were analyzed using the one-way ANOVA. All data were generated in three replicates. *P* < 0.05 was considered statistically significant.


**Ethics approval and consent to participate:** This research study was approved by the Institutional Review Board of First School of Clinical Medicine College.

## 3 Results

m6A methylation is involved in the transport, shearing, translation, and processing of mRNA. Its abnormality leads to changes in the corresponding downstream target proteins and the occurrence of human diseases. Here we detected the expression of methyltransferases and demethylases in TNF-α-treated HUVECs. Downregulated ALKBH5 was observed in TNF-α-induced inflammatory HUVECs. In the present research, we proved that ALKBH5 inhibited TNF-α-induced apoptosis through Bcl-2 pathway in HUVECs.

### 3.1 ALKBH5 was downregulated in TNF-α-treated HUVECs

**Figure 1:**
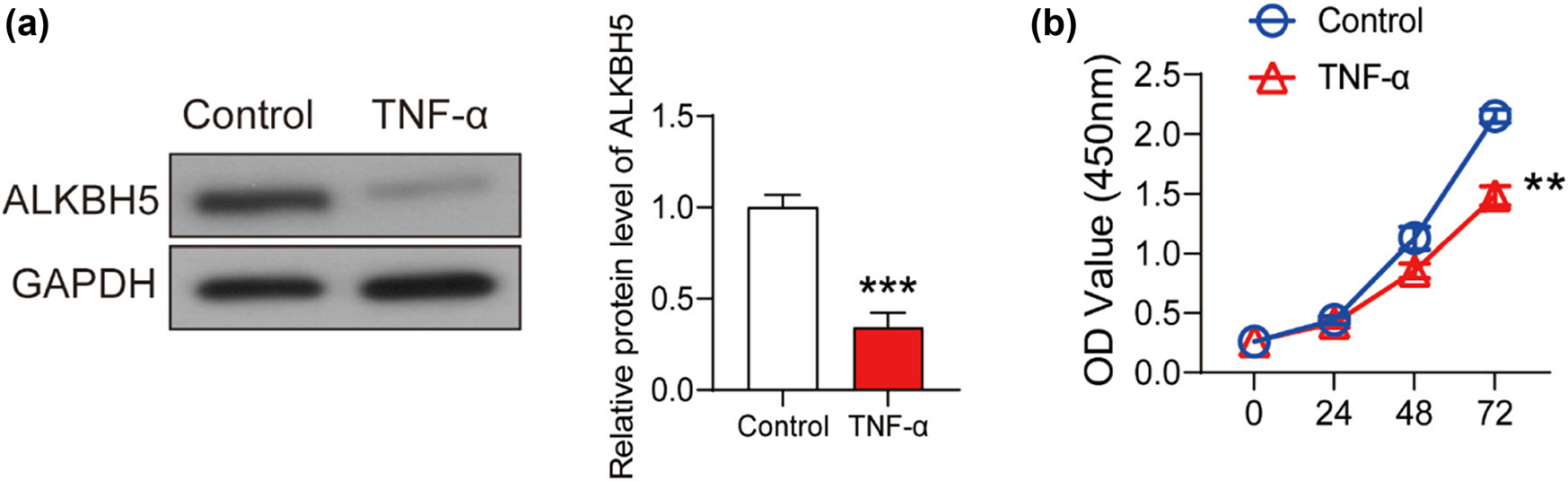
The model of vascular endothelial cell injury was constructed using TNF-α. (a) HUVECs were treated with 10 ng/mL TNF-α for 24 h, and cell viability was detected using CCK8 assay. PBS buffer was used as the control. The expression of ALKBH5 in TNF-α and control groups after the treatment for 24 h was analyzed using western blot. (b) Cell viability was detected using CCK8 assay. **P < 0.01; ***P < 0.001.

HUVECs were treated with 10 ng/mL TNF-α for 24 h to generate the vascular endothelial cell injury model (Figure 1a). As shown in Figure 1b, cell viability of HUVECs treated with TNF-α for 24 h declined significantly compared with that of the PBS-treated HUVECs. Based on this model, we investigated the specific effect of ALKBH5 on vascular endothelial cell injury.

To determine the effect of cell injury on ALKBH5 expression, the protein was extracted from HUVECs after the treatment of 10 ng/mL TNF-α for 24 h. As shown in Figure 1b, ALKBH5 was downregulated significantly after TNF-α treatment. Therefore, we further explored whether abnormal expression of ALKBH5 might affect HUVECs injury induced by TNF-α.

### 3.2 ALKBH5 attenuated the cell injury induced by TNF-α in HUVECs

**Figure 2:**
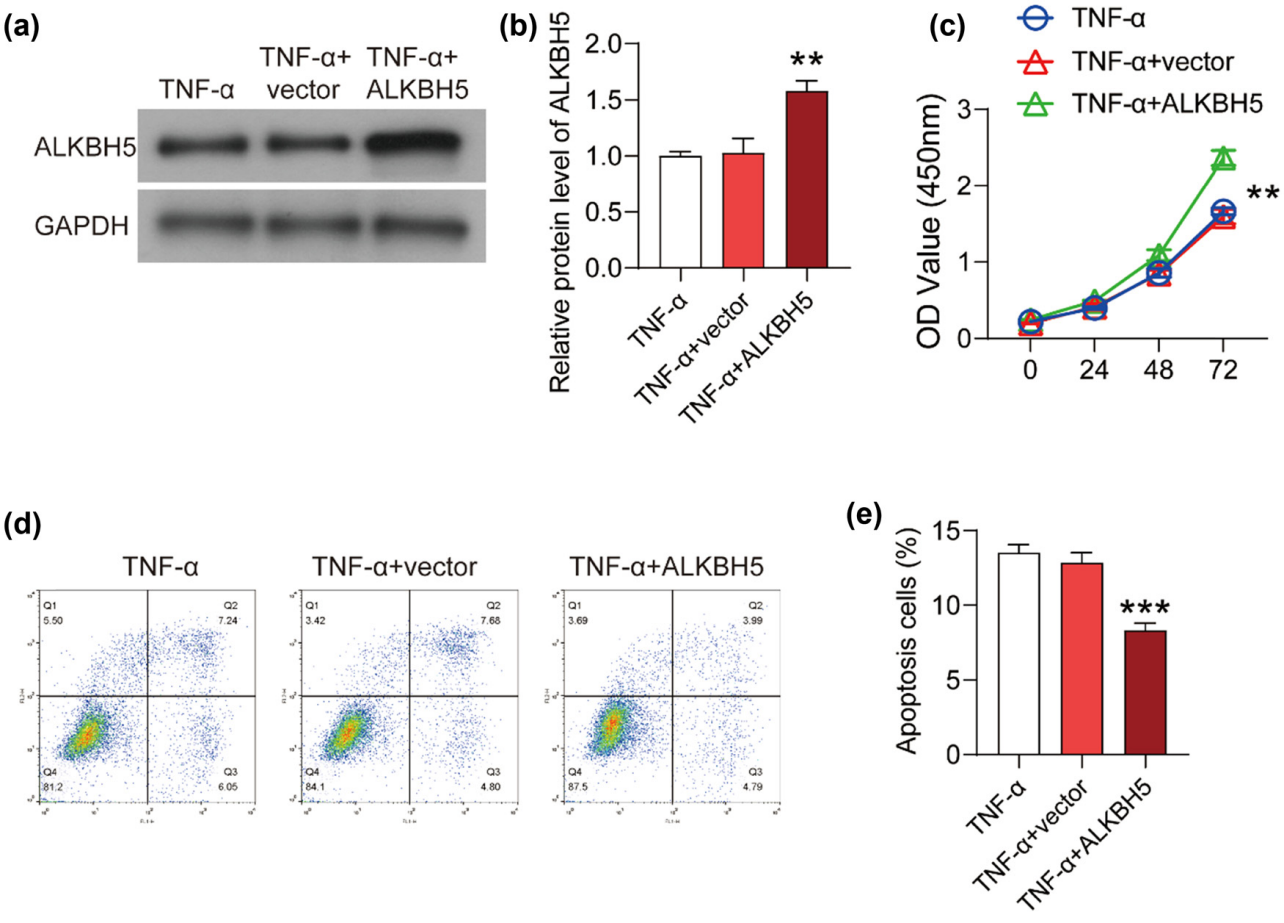
ALKBH5 attenuated the cell injury induced by TNF-α in HUVECs. (a) The overexpression plasmid of ALKBH5 was transfected into TNF-α-treated HUVECs with the empty plasmid as a negative control. Western blot was performed to detect the ALKBH5 protein level. (b) Relative protein level was analyzed using Image J software. (c) Cell viability was detected using CCK8 assay. (d) Flow cytometry analysis was preformed to detect the apoptosis of HUVECs. (e) The percentage of apoptotic cell number was analyzed using flowJo software. **P < 0.01; ***P < 0.001.

To investigate the specific effect of ALKBH5 on vascular endothelial cell injury, the overexpression plasmid of ALKBH5 was transfected into TNF-α-treated HUVECs with the empty plasmid as a negative control and TNF-α-treated HUVECs as the blank control. Western blot was performed to detect the level of ALKBH5. As shown in Figure 2a and b, compared with the empty plasmid, ALKBH5 overexpression plasmid significantly promoted the expression of ALKBH5. Then, we detected cell viability in these three groups. As shown in Figure 2c, cell viability indicated by OD450 increased significantly in HUVECs transfected with ALKBH5 plasmid. Results of flow cytometry for the apoptosis showed the opposite trend (Figure 2d). Compared to the negative control, the percentage of apoptotic cells in high ALKBH5 cells decreased significantly (Figure 2e). These data indicated that ALKBH5 might have a protective effect on TNF-induced cell injury in HUVECs.

### 3.3 Bcl2 blocked TNF-α-induced apoptosis by inhibiting the activity of genes downstream of p53

**Figure 3:**
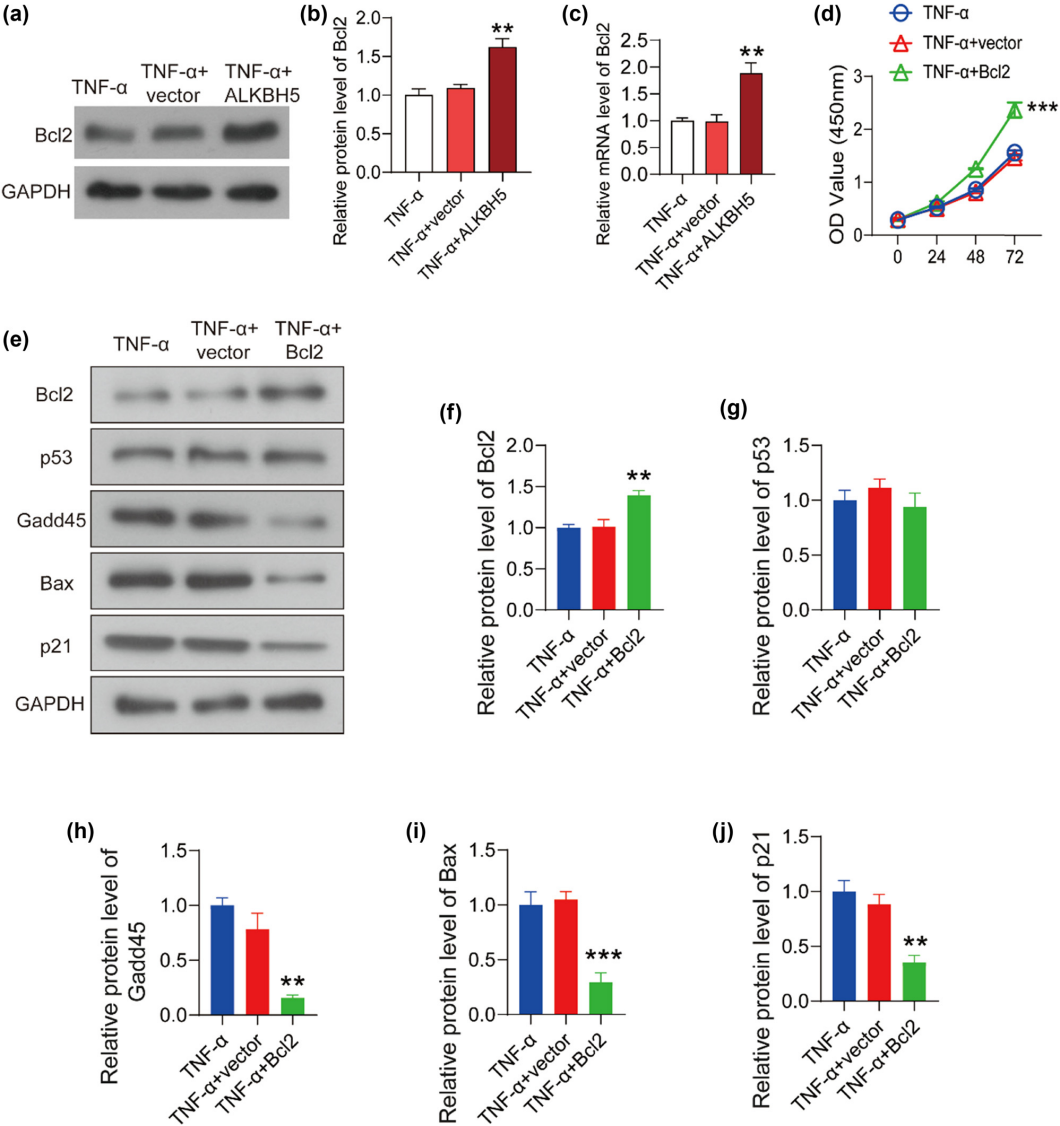
Bcl2 blocked the apoptosis induced by TNF-α through inhibiting transcriptional activity of p53. (a) The expression of Bcl2 was determined by western bolt in TNF-α treated HUVECs. (b) The relative protein level was analyzed using Image J software. (c) The mRNA level of Bcl2 was detected using qPCR. (d) The overexpression plasmid of Bcl2 was transfected into TNF-α-treated HUVECs with the empty plasmid as a negative control. Cell viability was detected using CCK8 assay. (e) The expression levels of apoptosis relative protein were detected using western blot. The protein levels of Bcl2 (f), p53 (g), Gadd45 (h), Bax (i), p21 (j) were analyzed using Image J software. **P < 0.01; ***P < 0.001.

Previous studies have shown that ALKBH5 inhibits Bcl2 degradation by reducing m6A modification, thereby regulating cell apoptosis in ovarian cancer, hepatocellular carcinoma, and cerebral ischemia-reperfusion injury [17,18,19]. To explore the mechanisms by which ALKBH5 inhibited the injury induced by TNF-α, we detected the effect of ALKBH5 on Bcl2 expression. As shown in Figure 3a and b, Bcl2 expression increased significantly after the overexpression of ALKBH5 in TNF-α-treated HUVECs. Importantly, the mRNA level of Bcl2 also increased markedly in ALKBH5 overexpressed cells, suggesting that ALKBH5 may block the degradation of Bcl2 mRNA through demethylation in HUVECs (Figure 3c).

To investigate the pathway by which Bcl2 inhibits the apoptosis, Bcl2 overexpression plasmid was transfected into HUVECs. As shown in Figure 3c, cell viability was enhanced after the overexpression of Bcl2. Then, the expression levels of apoptosis relative protein were determined using western blot (Figure 3d). As predicted, Bcl2 expression was upregulated after the transfection of Bcl2 overexpression plasmid (Figure 3e). Interestingly, there was no significant change in the expression of p53 after Bcl2 overexpression (Figure 3f). However, the expression levels of p53 transcription targets decreased significantly after Bcl2 overexpression, including Gadd45, Bax, and p21 (Figure 3g–i). These results indicated that Bcl2 attenuated TNF-α-induced cell injury through inhibiting the expression of p53 downstream targets.

### 3.4 ALKBH5 inhibits TNF-α-induced apoptosis of HUVEs via regulating Bcl2 expression

**Figure 4:**
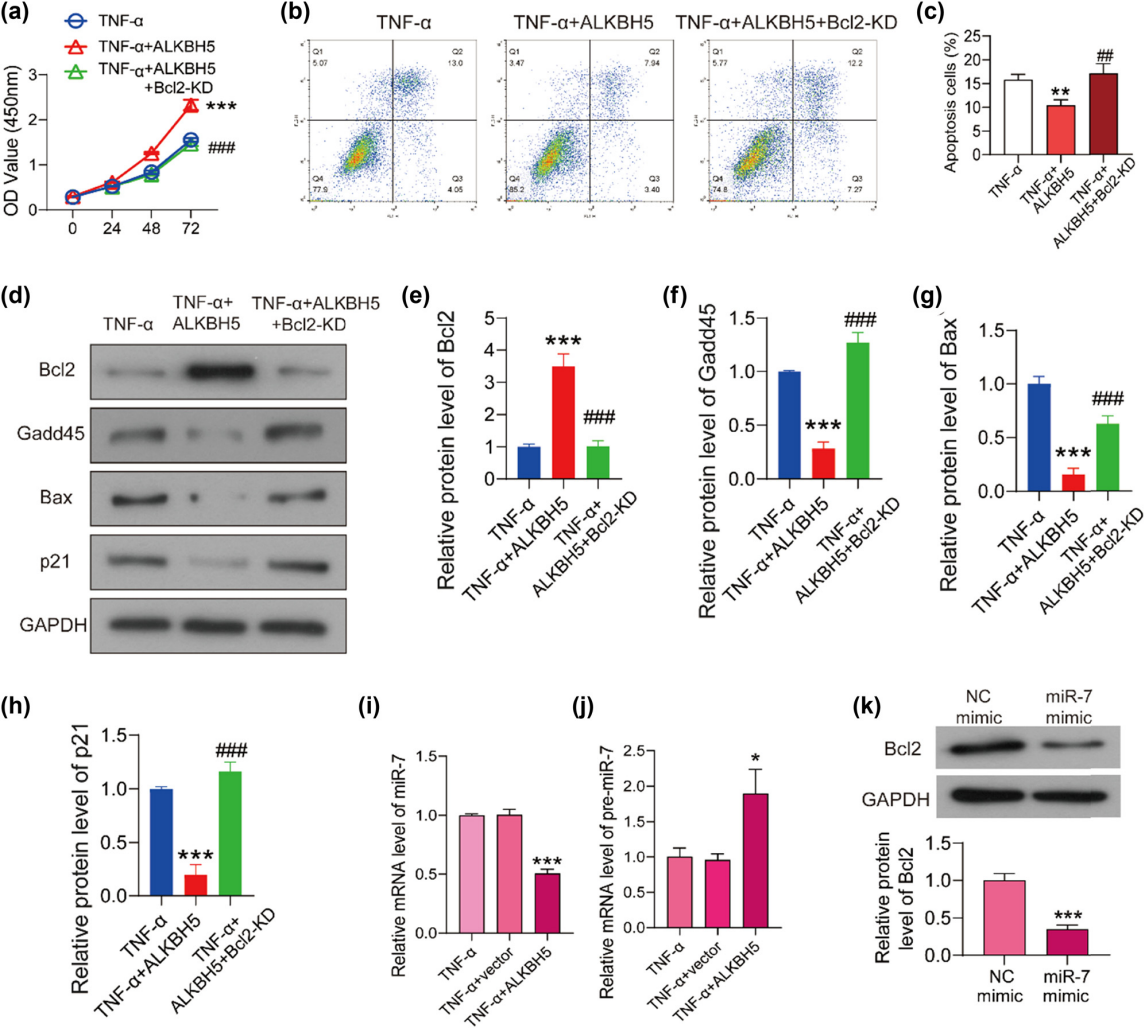
ALKBH5 inhibits TNF-α-induced apoptosis of HUVEs through regulating the expression of Bcl2. (a) The overexpression plasmids of ALKBH5 and Bcl2-siRNA were transfected into TNF-α treated HUVECs. Cell viability was detected using CCK8 assay. (b) Flow cytometry analysis was preformed to detect the apoptosis of HUVECs. (c) The percentage of apoptotic cell number was analyzed using flowJo software. (d) The expression levels of apoptosis relative protein were detected using western blot. The protein levels of Bcl2 (e), Gadd45 (f), Bax (g), and p21 (h) were analyzed using Image J software. The levels of miR-7 (i) and pri-miR-7 (j) were detected using qPCR. (k) negative or miR-7 mimic was transfected into HUVECs, and Bcl2 expression was detected using western blot. *P < 0.05 vs TNF-α + ALKBH5 group; **P < 0.01 vs TNF-α + ALKBH5 group; ***P < 0.001 vs TNF-α + ALKBH5 group; ^##^ *P* < 0.01 vs TNF-α + ALKBH5 + Bcl2 group; ^###^ *P* < 0.001 vs TNF-α + ALKBH5 + Bcl2 group.

To verify the hypothesis proposed above, the overexpression plasmid of ALKBH5 and Bcl2-siRNA were co-transfected into TNF-α-treated HUVECs. As shown in Figure 4a, the optical density (OD) value increased significantly after ALKBH5 overexpression, while it decreased to the control level after knocking down Bcl2, suggesting that Bcl2 knockdown hindered the promotion effect of ALKBH5 on cell viability. Knockdown of Bcl2 could counteract the significant decrease in apoptotic cell ratio caused by ALKBH5, and maintain it at the control level (Figure 4b and c). From the results of western blot, Gadd45, Bax, and p21 were significantly inhibited by ALKBH5, but the effect disappeared when Bcl2 was knocked down (Figure 4d–h). These results indicated that ALKBH5 inhibits TNF-α-induced apoptosis of HUVECs via regulating Bcl2 signaling pathway.

### 3.5 ALKBH5 inhibits the maturation of miR-7 in HUVECs

Previous study has shown that ALKBH5 promoted the expression of Bcl2 through regulating miR-7 in ovarian cancer [18]. Therefore, we detected the levels of miR-7 and pri-miR-7 in ALKBH5 overexpressed HUVECs by qPCR. As shown in Figure 4i and j, after ALKBH5 overexpression, miR-7 level increased significantly, while the level of pri-miR-7 decreased significantly compared with the control cells. Then, miR-7 mimic was transfected into HUVECs with the negative mimic as the control. As shown in Figure 4k, Bcl2 expression was significantly inhibited by miR-7 mimic. Combined with previous reports, we hypothesized that ALKBH5 might regulate the maturation of miR-7 through m6A modification, thereby regulating the expression of Bcl-2.

## 4 Discussion

In the present research, we investigated the specific role of demethylase ALKBH5 on TNF-α-induced vascular endothelial injury. ALKBH5 was identified as an RNA demethylase in 2013 [20,21]. It belongs to the α-ketoglutarate-dependent dioxygenase family and catalyzes the demethylation of m6A in a Fe(ii) and α-ketoglutarate-dependent manner. The modification and regulation of mRNA by M6A involves a variety of cellular processes, including mRNA splicing, export, localization, translation, and stability [22]. When methyltransferase is absent or demethylase is abnormal, it can affect cell development, differentiation, and metabolism. Therefore, the abnormality of m6A modification mechanism is related to a variety of human diseases [22]. Studies have shown that m6A methylation is relatively active in cardiomyocytes. In primary rat cardiomyocytes, 3,922 signal peaks of m6A methylation modification were detected [23]. In normal mouse myocardium, 24% of the transcripts can be detected with m6A methylation modification signals [24]. M6A methylation has been found to be closely related to common heart diseases such as cardiac hypertrophy, heart failure, and arrhythmia [24,25,26]. Importantly, m6A methylation is also associated with myocardial ischemic damage and hereditary heart disease [12,17,27].

According to our results, the expression level of demethylase ALKBH5 was significantly decreased in TNF-α-treated HUVECs. Importantly, ALKBH5 overexpression promoted proliferation and inhibited the apoptosis in TNF-α-treated HUVECs, suggesting that ALKBH5 could protect cells from TNF-α-induced cell injury. At present, there are few reports on the role and mechanism of m6A methylation related genes in AS. The expression of METTL14 and m6A level is significantly upregulated in atherosclerotic vascular endothelial cells (ASVEC). METTL14 promotes cell viability through promoting the processing of mature miR-19a [14]. In an analysis between m6A-associated single nucleotide polymorphisms (SNP) and blood pressure, approximately 10% of the blood pressure-associated m6A SNPs are correlated with stroke or coronary artery disease [28]. In another analysis, coronary artery disease (CAD)-associated m6A-SNPs are identified [29].

We further found that ALKBH5 could promote the expression of Bcl-2 in HUVECs. At the same time, high expression of Bcl2 reduced the expression of GADD45, Bax, and p21, which are transcriptionally activated by p53, but the expression of p53 has not been significantly affected. Based on the above results, we hypothesized that Bcl2 can regulate apoptosis by inhibiting the transcriptional activation of p53. However, there is no more evidence that the transcriptional activity of p53 is directly inhibited by ALKBH5, which still needs further experimental research. Several studies have shown that ALKBH5 inhibits Bcl2 degradation by reducing m6A modification, thereby regulating cell apoptosis in ovarian cancer, hepatocellular carcinoma and cerebral ischemia-reperfusion injury [17,18,19]. However, the specific pathway of this effect remains to be explored.

In the present research, we also explored the mechanism by which ALKBH5 promotes the expression of Bcl2. Previous studies have shown that ALKBH5 can regulate the expression of Bcl2 through miR-7, but the specific mechanism is still unclear [18]. In this study, we detected the levels of miR-7 and pri-miR-7 after ALKBH5 overexpression, and the results showed that ALKBH5 overexpression significantly increased the level of pri-miR-7 and decreased the level of miR-7. M6A-modified pri-miRNA can be recognized and processed by RNA-binding proteins, resulting in a decrease in mature miRNA expression and an increase in unprocessed pri-miRNA [30,31]. Therefore, we hypothesized that ALKBH5 may inhibit miR-7 maturation through m6A modification, thereby promoting Bcl2 levels. But this hypothesis needs further experimental confirmation.

## 5 Conclusion

ALKBH5 was downregulated in TNF-α-treated HUVECs and attenuated the TNF-α-induced cell injury through promoting the expression of Bcl2. This research expands our understanding of the progression mechanism of AS and provides potential treatment strategies for AS.
